# Arm crossing updates brain functional connectivity of the left posterior parietal cortex

**DOI:** 10.1038/srep28105

**Published:** 2016-06-15

**Authors:** Hiroki Ora, Makoto Wada, David Salat, Kenji Kansaku

**Affiliations:** 1 Systems Neuroscience Section, Department of Rehabilitation for Brain Functions, Research Institute of National Rehabilitation Center for Persons with Disabilities, Tokorozawa 359-8555, Japan; 2 Brain Science Inspired Life Support Research Center, The University of Electro-Communications, Chofu 182-0021, Japan; 3 Department of Computational Intelligence and Systems Science, Tokyo Institute of Technology, Yokohama 226-8503, Japan; 4 Athinoula A. Martinos Center for Biomedical Imaging, Department of Radiology, Massachusetts General Hospital, Charlestown, MA 02129, USA

## Abstract

The unusual configuration of body parts can cause illusions. For example, when tactile stimuli are delivered to crossed arms a reversal of subjective temporal ordering occurs. Our group has previously demonstrated that arm crossing without sensory stimuli causes activity changes in the left posterior parietal cortex (PPC) and an assessment of tactile temporal order judgments (TOJs) revealed a positive association between activity in this area, especially the left intraparietal sulcus (IPS), and the degree of the crossed-hand illusion. Thus, the present study investigated how the IPS actively relates to other cortical areas under arms-crossed and -uncrossed conditions by analyzing the functional connectivity of the IPS. Regions showing connectivity with the IPS overlapped with regions within the default mode network (DMN) but the IPS also showed connectivity with other brain areas, including the frontoparietal control network (FPCN). The right middle/inferior frontal gyrus (MFG/IFG), which is included in the FPCN, showed greater connectivity in the arms-crossed condition than in the arms-uncrossed condition. These findings suggest that there is state-dependent connectivity during arm crossing, and that the left IPS may play an important role during the spatio-temporal updating of arm positions.

Appropriate motor execution requires coherent neural representations of the configuration of body parts as they are localized in external space^1^,^2^. The unusual configuration of body parts has been reported to cause several types of illusions^3^,^4^,^5^. In particular, when a pair of tactile stimuli is delivered to crossed arms (one to each hand) a reversal or confusion of subjective temporal ordering occurs^6^,^7^,^8^.

Our research group has reported that the crossing of one’s arms in the absence of any external sensory stimuli causes increased activation of the blood oxygen level-dependent (BOLD) signal in the left posterior parietal cortex (PPC)^9^. Furthermore, an assessment of tactile temporal order judgments (TOJs) in the same individuals during a functional magnetic resonance imaging (fMRI) scan revealed a positive association between activity in this area and the degree of reversal/confusion in subjective temporal ordering due to arm crossing. In this case, the strongest positive association with the reversal or confusion was observed in the left intraparietal sulcus (IPS)^9^.

Previous studies have highlighted the involvement of the superior aspect of the PPC during the neural processing of bodily information^10^. Lloyd *et al*.^11^ demonstrated that when the right hand is placed on the opposite side of the body across the midline with the eyes open there is increased BOLD activity in the left ventral intraparietal (VIP) sulcus. It has also been reported that the left superior parietal lobule (SPL) is activated during the updating of limb positions^12^. Furthermore, a recent study investigating the electrophysiological correlates of tactile remapping, which combines somatosensory and proprioceptive information, observed a strong left-sided lateralization^13^, which is consistent with previous findings from our research group^9^.

Functional imaging techniques have allowed researchers to estimate functional connectivity^14^ using spontaneous BOLD activity^15^,^16^. For example, Smith *et al*.^17^ showed that the functional connectivity pattern during a resting state corresponded to the co-activation pattern during the task itself. Yeo *et al*.^18^ used a clustering approach to identify seven large-scale cerebral networks based on the intrinsic functional connectivity of 1,000 subjects, and suggested that these reliable networks may reflect anatomical connectivity. Buckner *et al*.^19^ also suggested that functional connectivity is not a simple proxy for static anatomical connectivity and recent studies have begun to demonstrate that functional connectivity can be altered by brain maturity^20^,^21^, training, including perceptual learning^22^ and motor learning^23^,^24^,^25^, subject-driven cognitive states^26^, and the level of consciousness while under anesthesia^27^. Based on these observations, these authors^19^ proposed that functional connectivity MRI (fcMRI) results reflect the combination of a stable anatomically constrained component and a state-dependent signal component.

Previous positron emission tomography (PET) studies have suggested that the bilateral PPC and the dorsolateral frontal cortex (dlPFC) support the neural representation of body parts^28^,^29^. In fact, as described above, our research group reported that the crossing of one’s arms causes increased BOLD activation in the left PPC^9^. Taken together, these results suggest that, when activated by arm crossing, the left PPC is responsible for the neural representation of the configuration of body parts. Therefore, a functional connectivity analysis of the left IPS may provide clues to understanding how the left IPS collaborates with other brain areas to represent the configuration of body parts.

In the present study, functional connectivity analyses were applied to examine the functional anatomy of the left IPS when it was activated by arm crossing. The results indicated functional connectivity between the left IPS and the right middle frontal gyrus or inferior frontal gyrus (MFG/IFG) that exhibited an increase during arm crossing.

## Results

**Figure 1:**
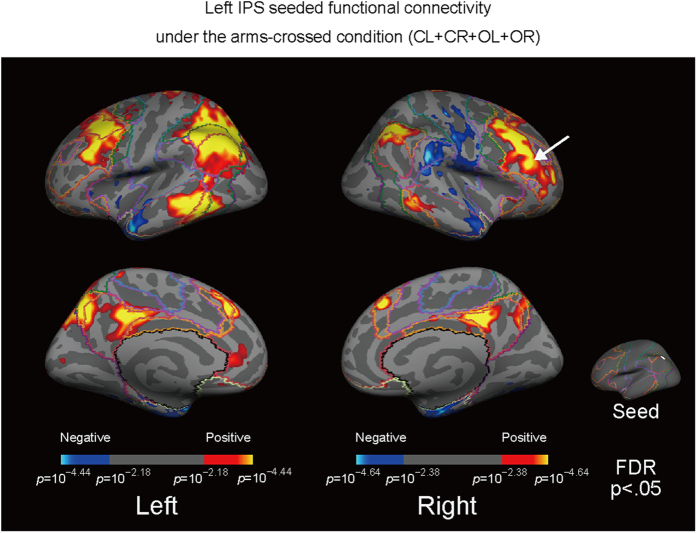
Under the arms-crossed condition, regions showing connectivity with the left IPS overlapped with regions in the DMN but the left IPS also showed connectivity with other brain areas including the right dlPFC. The superimposed colored boundary (annotation) is the estimated “seven networks” of the brain from Yeo *et al*.^18^; (purple: visual; blue: somatomotor; green: dorsal attention; violet: ventral attention; cream: limbic; orange: frontoparietal; red: default).

**Figure 2:**
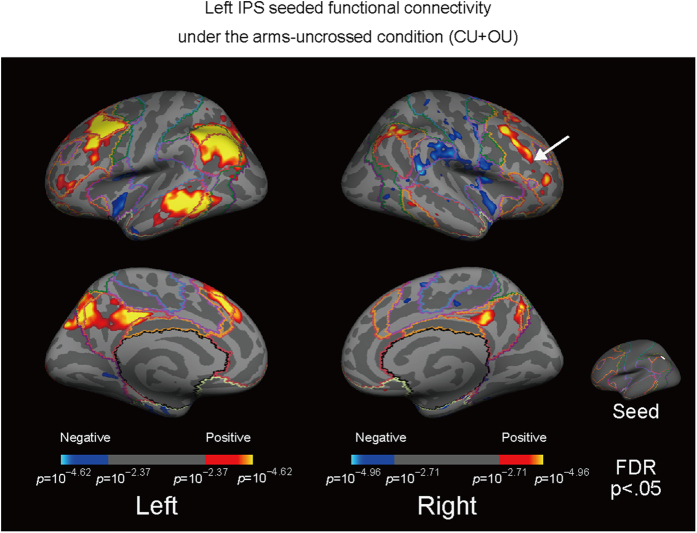
Under the arms-uncrossed condition, regions showing connectivity with the left IPS overlapped with regions in the DMN but the left IPS also showed connectivity with other brain areas including the dlPFC. The superimposed colored boundary (annotation) is the estimated “seven networks” of the brain from Yeo *et al*.^18^; (see the legend of Fig. 1).

The present study examined the functional connectivity between the left IPS and the rest of the brain during test epochs under an arms-crossed condition (Fig. 1) and an arms-uncrossed condition (Fig. 2). In a previous study from our group^9^, participants (n = 20) were instructed to change their arm position from a resting position (outstretched beside the legs) to a test position (on the legs) with their arms either uncrossed or crossed during each epoch, as cued by auditory beeps, while in a MR scanner. The participants’ eyes were either closed (C) or open (O) and each participant had three arm positions: left arm over right (L), right arm over left (R), and arms uncrossed (U). After scanning, participants took part in a tactile TOJ task and the associations between BOLD activity and the subjective reversal or confusion during the TOJ task were examined. In the present study, the seed region that exhibited the strongest correlation with the reversal of subjective temporal order was adopted for the functional connectivity analysis (n = 24, all subjects were male, age range: 19–44 years), which was the same method used in our previous study^9^. The seed region was the left IPS (−37, −60, 48; in Talairach coordinates) and covered a circle approximately 10 mm in radius.

The factors eyes C and eyes O and two types of crossing (L and R) were not included in the analysis, as we focused on the effect of arm crossing. The crossed-hand conditions contain double the number of trials compared to the uncrossed condition. We used Bartlett’s theory to address the effect of differences in the number of trials.

The left IPS-seeded functional connectivity map overlapped with the default mode network (DMN) under both the crossed and uncrossed conditions (Figs 1 and 2). Additionally, the left IPS showed connectivity with other brain areas including the right dlPFC (Figs 1 and 2), which has been reported to be anticorrelated with the DMN^16^. Additionally, the numbers of vertices were counted to evaluate any overlapping areas. There were 15,106 vertices that overlapped with the DMN in the left hemisphere and 5,262 vertices that overlapped with the DMN in the right hemisphere, which is the ipsilateral side to the seed region (left IPS). Additionally, 8,937 vertices in the left hemisphere and 9,928 vertices in the right hemisphere overlapped with the FPCN.

**Figure 3:**
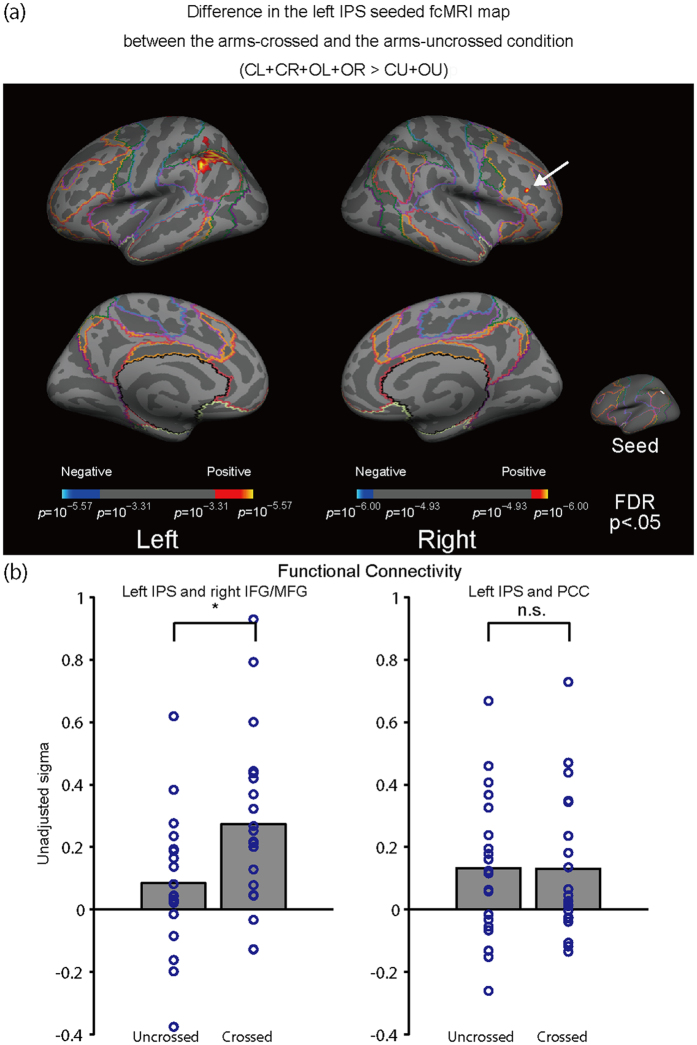
(**a)** Arm crossing updated the left IPS-seeded fcMRI map. During the arms-crossed condition, functional connectivity of the left IPS became stronger in the right middle/inferior frontal gyrus (MFG/IFG; a white arrow; the area is also marked in Figs 1 and 2). The superimposed colored boundary (annotation) is the estimated 7-network from Yeo *et al*.^18^ (see the legend of Fig. 1). (**b) (**Left-hand panel) The right dlPFC (from Vincent *et al*.^30^), which is included in the FPCN, showed a greater intensity of connectivity to the left IPS under the arms-crossed condition than under the arms-uncrossed condition (*P*_FDR_ < 0.05). An unadjusted sigma indicates the Z-values that were not adjusted for sample size. (Right-hand panel) The posterior cingulate cortex^56^, which is included in the DMN, did not show updating with the PPC under either the arms-crossed or arms-uncrossed conditions. An unadjusted sigma indicates the Z-values that were not adjusted for sample size.

To evaluate differences in functional connectivity between the arms-crossed and the arms-uncrossed conditions, the differences in the functional connectivity between the left IPS and the rest of the brain were assessed. The difference in functional connectivity between the arms-uncrossed and the arms-crossed conditions are show in Fig. 3a (*P*_*FDR*_ < 0.05). During the arms-crossed condition, the functional connectivity of the left IPS became stronger in the right MFG/IFG and stronger connectivity was also identified in the left PPC (Table 1). Figure 3b illustrates the difference in functional connectivity between the arms-crossed and arms-uncrossed conditions extracted from Fig. 3a. The right MFG/IFG, which is included in the FPCN^30^, showed a greater functional connectivity to the left IPS under the arms-crossed condition than under the arms-uncrossed condition (Fig. 3b; *P*_*FDR*_ < 0.05). Furthermore, the posterior cingulate cortex (PCC), which is included in the DMN^31^, did not respond to the updated functional connectivity to the left IPS under either the arms-crossed or arms-uncrossed conditions (Fig. 3b).

## Discussion

In the present study, the functional anatomy of the left IPS during activation, induced by the crossing of one’s arms was examined using functional connectivity techniques. The findings demonstrated that the functional connectivity between the left IPS and the right MFG/IFG exhibited an increase during arm crossing.

### Changes in the functional connectivity of the left IPS during arm crossing

The present study observed an increase in functional connectivity between the left IPS and the right MFG/IFG due to arm crossing. Recent studies have demonstrated that functional connectivity can be altered by perceptual learning^22^ or motor learning^23^,^24^,^25^ as well as by subject-driven cognitive states^26^. The present findings indicate that functional connectivity can also be altered by changing the posture of one’s arms. Buckner *et al*.^21^ suggested that intrinsic functional connectivity reflects a combination of stable anatomical connectivity and state-dependent signal components. The present data may have captured a state-dependent signal component.

There are robust anatomical connections extending between the PPC and the frontal cortex. Electrophysiological recordings in macaques have shown that neurons in the VIP area respond to both visual and tactile stimulation^32^, much like neurons in the ventral premotor cortex respond to both visual and tactile stimuli^33^. Taken together, these findings suggest that both the PPC and the frontal cortex play a role in the updating of spatial coordinates and posture. It has also been proposed that the right MFG is a crucial area within the spatial working memory network^34^ and that the right inferior frontal cortex (IFC) is responsible for inhibition^35^. It is possible that the functional connectivity of the left IPS that was observed in the present study may be switched with that of the right IFC in a top-down manner due to arm crossing.

### Functional role of the left IPS

The present findings indicate that the left IPS, which is associated with an increase in reversals resulting from arm crossing^9^, was functionally connected with cortical regions that overlap with the DMN and with the FPCN^30^. Furthermore, these findings demonstrate that the right MFG/IFG, which is included in the FPCN, showed greater intensity of functional connectivity under the arms-crossed condition than under the arms-uncrossed condition. On the other hand the PCC, which is included in the DMN, did not show greater intensity of functional connectivity with the left IPS region due to arm crossing.

When a human is doing “nothing”, brain activities in the cerebral midline and lateral cortical regions increase^31^. These brain regions are considered to be parts of the DMN^31^, which has been investigated using PET and fMRI^31^ scans as well as magnetoencephalograms^36^. Based on the findings of BOLD fMRI studies, it has been proposed that activity in the DMN is suppressed during attention to the external world^31^ and that this activity is associated with internal mentation^37^,^38^. This is an interesting contrast with the activity within an antagonistic network known as the “task positive network” (TPN) that is related to externally directed cognition^39^. A previous study using an analysis based on step-wise functional connectivity MRI scans^40^ showed that the DMN is located most distant from the low-level sensory areas of the cortex^40^ and proposed that the DMN is associated with autobiographical information, the self, and social functions^41^. These findings suggest that the DMN is associated with the perception of self.

The present study also found that the left IPS was functionally connected with other brain areas, including the FPCN, which is anticorrelated with the DMN^16^ and may be involved in cognitive control and decision-making processes^30^. Thus, the present results indicate that the left IPS may play a role as a gateway that connects the DMN to other brain areas, including the FPCN.

### Spatio-temporal updating in the human brain

The left IPS, which is included in the PPC, may be responsible for the neural representations of the position or configuration of body parts; this has been termed “body schema”^1^,^2^. The left IPS may also play an important role in the confusion/reversal that is associated with arm crossing during a TOJ task^9^,^13^ and may be responsible for neural representations that overlap with the representation of peripersonal space^42^. Furthermore, as suggested above, the left IPS may be associated with the self due to its inclusion in the DMN. Previous fMRI studies have observed abnormalities in the DMNs of individuals with autism^43^ and our research group recently reported that autism is associated with a low degree of confusion/reversal due to arm crossing during the TOJ task^44^ such that autistic children demonstrate significantly less illusory confusion/reversal than neurotypical children. Furthermore, young boys with higher autism spectrum quotient (AQ) scores generally show less crossed-hands confusion/reversal^44^. Because confusion/reversal is known to be acquired in early childhood^45^, the former study discussed that rudimentary spatio-temporal processing of tactile stimuli may persist in individuals with autism^44^.

Confusion/reversal due to the crossing of one’s arms may be caused by the conflicting influences of multiple frames of ref. 6. Furthermore, the change in functional connectivity within the putative neural network that supports the neural representation of body parts localized in external space, which was estimated with a seed-based functional connectivity analysis of the left PPC, may be related to the modulation of the neural representations of frames of reference. Further investigation may provide clues to fully understanding spatio-temporal updating in the human brain.

## Methods

The present study was approved by the institutional ethics committee at the National Rehabilitation Center for Persons with Disabilities and all participants provided written informed consent according to institutional guidelines. All experiments were carried out in accordance with the approved guidelines. In the present study, a seed-based functional connectivity analysis was applied along the cortical surfaces, which were reconstructed using the Freesurfer software package (MGH, Harvard Medical School, Boston, USA). The preprocessing of the fcMRI analyses was the same as in past fMRI studies but a surface-level analysis similar to that of Yeo *et al*.^18^ was applied. Thus, the later steps of the preprocessing differed from those of previous studies^15^,^16^,^30^.

### Participants

The present study included 24 participants (all males, age range: 19–44 years); 20 of the participants also participated in a previous study by our research group^9^. Only male participants were recruited for this study because sex differences in the magnitude of the paradoxical experiences elicited when performing tactile temporal order judgments with crossed hands have been previously reported^46^. All the participants were neurologically normal and strongly right-handed (+60 ≤ LQ ≤ +100) according to the Edinburgh Inventory^47^.

### MR scanner task

Each participant was placed in a MR scanner with his or her arms uncrossed under one condition and crossed under the other condition. The participants’ eyes were either closed (C) or open (O) and each participant had three arm positions: left arm over right (L), right arm over left (R), and arms uncrossed (U). As a result, each participant experienced six conditions in total with the order of presentation counterbalanced across participants. Each condition consisted of four 40-second epochs; different auditory beeps were used to mark the start and end of each epoch. Participants were instructed to change their arm position from the resting position (outstretched beside the legs) to the test position (on the legs) with their arms either uncrossed or crossed (Crossed L or Crossed R) during each epoch. Prior to the task, the participants were verbally instructed regarding the task contents and practiced the tasks several times. During the experiments, the participants’ ears were plugged to reduce background noise and the auditory beeps and instructions were delivered through earphones (Avotec SilentScan SS3000; Avotec, Inc., Stuart, FL, USA). The participants’ movements were visually monitored from an operator’s room through a window on the foot side of the scanner.

### Scanning parameters

All functional MRI data were acquired with a 1.5 Tesla MRI scanner (Toshiba Medical Systems; Tochigi, Japan). Functional images sensitive to BOLD contrast were obtained from a T2* gradient-echo echo-planar imaging pulse sequence with 220 mm field-of-view, 6 mm slice thickness, 2 mm interslice gap, and a 64 × 64 data matrix. For each session, 180 image volumes were acquired with a TR of 2000 ms, a TE of 40 ms, and a flip angle of 85°. The image volumes covered the entire brain with 20 slices.

### Functional data preprocessing

The functional data were preprocessed with a series of steps common to fMRI analyses as follows: 1) removing the first four volumes, 2) compensating for slice time correction using SPM8 (Wellcome Department of Cognitive Neurology; London, UK), and 3) correcting for head motion using six degrees-of-freedom rigid-body registration with the FSL package^48^,^49^.

Additionally, further preprocessing steps were performed to optimally condition the fMRI data for a functional connectivity analysis. A band-pass filter was applied to retain data from 0.01–0.08 Hz and several sources of spurious variance were then removed from the functional data through linear regression: 1) the six parameters obtained by rigid-body correction of head motion, 2) the whole-brain signal averaged over fixed regions in atlas space, 3) signals from a ventricular region of interest, and 4) signals from a region centered in the white matter. No spatial smoothing of the functional data occurred up to this point in the preprocessing sequence.

### Structural MRI preprocessing and functional-structural image coregistration

All anatomical MRI data were processed using Freesurfer (stable-5-20110525 on Mac OSX 10.6.8, Mac Pro 5,1 [Mid 2010)) and the functional-structural image coregistration method was similar to that of Yeo *et al*.^18^. Through the coregistration process, the voxel-based functional data were finally transformed into surface-based (vertices-based) data.

### Functional connectivity analysis

Functional connectivity analyses of BOLD fMRI signals have contributed to the identification of brain areas that collaborate over the whole brain. A functional connectivity analysis can be roughly divided into two techniques (for a review, see^50^), seed-based^15^,^16^,^51^ and model-free (e.g., independent component analysis [ICA]-based^52^); the results of these techniques strongly overlap^50^.

A spatial filter (full width half maximum [FWHM] = 6 mm) was applied to the functional data of each subject on the template surface named “fsaverage”. Then, a seed region whose coordinates were taken from our previous study^12^ was adopted; the seed region was the left IPS (−37, −60, 48) and it covered a circle approximately 10 mm in radius. The mean time course of the seed region was used for a correlation analysis across all vertices distributed on the surface of the cortex (see^51^,^53^). In this analysis, the time course of the seed region, which was windowed with the HRF-convoluted boxcar model, was used (see^54^) to examine functional connectivity under the arms-crossed condition. The obtained correlation coefficients were converted into *z*-values with Fisher’s r-to-z transformation and Student’s two-tailed paired *t*-tests were used to determine functional connectivity. Each map was corrected for multiple comparisons at a significance level of *P*_FDR_ < 0.05.

### Differences in functional connectivity

To examine the differences in functional connectivity between the arms-crossed and arms-uncrossed conditions, the mean time course of the seed region (see^51^,^53^), which was weighted using the HRF-convoluted boxcar model, was used for the correlation analysis. A cluster-size threshold of 20 mm^2^ was applied to obtain a summary table (Table 1). The obtained correlation coefficients were converted into *z*-values with Fisher’s r-to-z transformation, correcting for degrees of freedom in accordance with Bartlett’s theory^16^ and Student’s two-tailed paired *t*-tests were used to detect differences in connectivity. We used Bartlett’s theory to address the effect of differences in the number of trials. Each map was corrected for multiple comparisons at a significance level of *P*_FDR_ < 0.05. Talairach Client^55^ was used to label differences in functional connectivity.

## Additional Information

**How to cite this article**: Ora, H. *et al*. Arm crossing updates brain functional connectivity of the left posterior parietal cortex. *Sci. Rep.* **6**, 28105; doi: 10.1038/srep28105 (2016).

## Figures and Tables

**Table 1:** Brain regions with significant differences in left IPS-seeded functional connectivity between the arms-crossed and arms-uncrossed conditions.

Peak coordinates			Region		t value	p value	Size (mm^2)
X	Y	Z					
38.5	30	14.3	Right Middle Frontal Gyrus	Brodmann area 46	6.29	<0.001	22.48
−36.5	−61.8	43.1	Left Inferior Parietal Lobule	Brodmann area 7	9.96	<0.001	1216.36
−26.7	−57.5	44.6	Left Superior Parietal Lobule	Brodmann area 7	4.56	<0.001	32.56
−34.6	−47.4	53.4	Left Superior Parietal Lobule	Brodmann area 7	4.35	<0.001	80.41
−11.8	−25	61.3	Left Medial Frontal Gyrus	Brodmann area 6	4.15	<0.001	25.55

Each map was corrected for multiple comparisons at a significance level of P_FDR_ < 0.05 with a cluster-size threshold of 20 mm^2^.
